# Using Multimodal Imaging to Correlate Link Between Intracranial Aneurysms and Acute Ischemic Stroke

**DOI:** 10.3390/diagnostics16101511

**Published:** 2026-05-16

**Authors:** Vania Anagnostakou, Patrick Thurner, Jawid Madjidyar, Miklos Krepuska, Anna Kyselyova, Tilman Schubert, Zsolt Kulcsar

**Affiliations:** 1 Department of Neuroradiology, Clinical Neuroscience Center, University Hospital Zürich, Frauenklinikstrasse 10, 8091 Zürich, Switzerland; patrick.thurner@usz.ch (P.T.);; 2 New England Center for Stroke Research, Department of Radiology, University of Massachusetts Chan Medical School, 55 N Lake Ave, Worcester, MA 01655, USA

**Keywords:** cerebral aneurysm, ischemic stroke, vessel wall imaging, thromboembolic events, advanced imaging

## Abstract

**Background:** The coincidence of unruptured cerebral aneurysms in acute ischemic stroke (AIS) is well known; however evidence on causality remains unclear. In this paper we are aiming to highlight key imaging characteristics that can aid in establishing or excluding a causative relationship between the two entities. **Methods:** Eight symptomatic patients with ischemic stroke and presence of an aneurysm in the same vascular territory were retrospectively analyzed. The patients were evaluated with computed tomography (CT) or magnetic resonance imaging (MRI) and depending on initial imaging findings, patients received either digital subtraction angiography (DSA) and MRI or MRI alone, with or without vessel wall imaging (VWI). Eligible patients received mechanical thrombectomy (MT) and the rest were managed conservatively. **Results:** The analysis of the imaging findings led to a proposed framework for classification/characterization of aneurysm as a possible, probable or improbable cause of AIS. The main findings used to categorize the aneurysmatic lesions were aneurysm thrombosis, positive vessel wall imaging, location and presence of comorbidities. Depending on the category the aneurysm was classified in, a decision regarding conducting treatment or not was made. **Conclusions:** Detailed observation of traditional imaging along with advanced MRI sequences like VWI can potentially help stratify the probability of aneurysms being the source of thromboembolic events.

## 1. Introduction

Unruptured cerebral aneurysms are frequently found in patients with acute ischemic stroke (AIS) and their detection is now possible due to the increased use of advanced neuroimaging modalities. Although the prevalence of such aneurysms in this patient cohort appears to be higher than in the general population [1,2], their presence does not necessarily correlate with or significantly alters the clinical outcome related to the ischemic event [3]. Furthermore, establishing a definitive relationship between unruptured aneurysms and stroke is challenging and difficult to prove [4,5].

Cerebral aneurysms in remote vascular territories can be classified as incidental or coincidental findings. However, aneurysms identified within the same vascular territory as the AIS may raise questions as to whether the lesion contributed to the etiology of stroke. Ischemic events may occur distal to both small and large unruptured intracranial aneurysms [6]. Large aneurysms may cause ischemia by mechanical compression or due to thrombus formation within the aneurysm sac. These thrombi may subsequently embolize into the distal cerebral arteries or may extend into the parent vessel, leading to partial or complete vascular occlusion and downstream ischemia [1,7,8]. In contrast, determining the causality in cases involving small aneurysms is far more difficult to address, due to small size, lack of compression and imaging limitations of intra-aneurysmal thrombosis. Additionally, the natural history of unruptured intracranial aneurysms presenting with ischemic symptoms remains largely undefined [9] and the clinical course and outcome of these patients is not well-documented.

Further complicating the identification of etiology is the frequent presence of other coexisting, potential causes of ischemic stroke, such as atrial fibrillation, large artery atherosclerosis and small vessel disease. Although causality between the aneurysm and AIS is difficult to establish, imaging findings may suggest a spectrum of likelihood for aneurysm-related stroke from highly probable to highly improbable. With this case series, including patients with concomitant unruptured cerebral aneurysm and ischemic stroke, we aim to draw attention to aneurysmal instability and emphasize the importance of meticulous neuroimaging analysis in evaluating aneurysm characteristics, as certain features may provide valuable diagnostic clues. A sophisticated understanding of these imaging findings may support more accurate risk stratification and help inform decisions regarding further management, surveillance, and potential intervention in select patients.

## 2. Materials and Methods

### 2.1. Patient Selection

We retrospectively analyzed eight patients with acute and subacute ischemic strokes who were admitted to our hospital, in the period between 2017 and 2023. All patients were harboring an intracranial aneurysm in the same vascular territory as their ischemic event. At presentation all patients were symptomatic with a neurologic deficit and corresponding ischemic lesions, evident either on computed tomography (CT) or magnetic resonance imaging (MRI). Aneurysms in this cohort were initially diagnosed using either MRI or digital subtraction angiography (DSA). In cases where patients presented with a large vessel occlusion (LVO), angiographic evaluation was performed during endovascular treatment with mechanical thrombectomy (MT). Aneurysm detection within the affected vascular territory was feasible, mainly after successful recanalization. All patients with acute vessel occlusion in the vicinity of the aneurysm or with downstream ischemic lesions in the same vascular territory were included in our analysis. Patient demographics, clinical characteristics, imaging work-up, imaging findings and treatment plan for each patient are presented in Table 1 and Table 2. The diagnostic imaging was analyzed by two non-blinded independent investigators (VA, ZK). This study was approved by the regional ethics board (Basec No: 2022-0042). Informed consent was obtained from all patients.

### 2.2. Imaging and Stroke Evaluation

**Figure 1:**
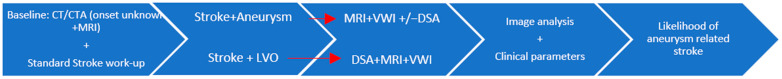
Diagnostic workflow.

All patients received a CT with CT angiography (CTA) at baseline as first-line imaging of their stroke work-up, except one patient who was initially evaluated with both MRI and CT/CTA due to inability to reliably establish symptom onset. Depending on initial imaging findings patients received either DSA and MRI or MRI alone, with or without vessel wall imaging (VWI). DSA was performed in all patients with LVOs, who were candidates for mechanical thrombectomy (MT), and in patients where further clarification of aneurysm characteristics was deemed necessary. VWI was performed in a 3-Tesla MRI, with the use of a high-resolution submillimeter 3D T1 SPACE black blood sequence without and with application of contrast medium, with a dose of 0.1 mmol/kg. All patients received the standard stroke work-up per the protocol of our institution, with assessment of the intra- and extracranial vessels, with additional Doppler ultrasound (US) of the extracranial vasculature when needed, evaluation of cardiac function with electrocardiogram (ECG) and transesophageal echocardiography (TEE) as well as a complete laboratory investigation. Symptoms and age of ischemic lesions at presentation are shown in Table 1. The correlation of the aneurysm with the ischemic stroke was evaluated based on aneurysm characteristics and other coexisting causes of stroke. Presence of thrombus in the aneurysm sac and aneurysm wall enhancement based on black blood imaging increased the probability of the correlation. Concerning stroke etiology, the presence of stroke risk factors, such as atrial fibrillation and large artery atherosclerosis, weakened the correlation. Taking these factors into consideration, we proposed a three-tiered classification system reflecting different diagnostic certainty levels, categorizing each case as probable, possible or improbable. The diagnostic workflow is shown in Figure 1.

### 2.3. Treatment

Patients with acute stroke and evidence of LVO on non-invasive imaging received intravenous (i.v.) thrombolysis when eligible and if within the acceptable standard therapeutic time window of 4.5 h. This was followed by MT. The choice of thrombectomy technique, whether stent-retriever thrombectomy, aspiration thrombectomy or a combination of both, was based on the interventionalist’s clinical judgment and preference, the location of the occlusion and anatomical characteristics. In cases where larger aneurysms were identified and were considered potentially unstable or with elevated rupture risk, definitive treatment options including endovascular coiling or surgical clipping were considered. Smaller aneurysms, which were considered low risk for bleeding at the time of diagnosis, were conservatively managed and closely monitored.

## 3. Results

Patient age ranged between 44 and 83 years (median age = 72.5, IQR 60.5–80) and all patients but one were female (F = 7, M = 1). The National Institute of Health Stroke Scale (NIHSS) at presentation ranged between 2 and 23 points (median NIHSS = 5.5, IQR 3–11). Patients with LVO had higher NIHSS, ranging between 23 and 10 points (median NIHSS = 11, IQR 7–17.5). Five patients were diagnosed with acute and three patients with subacute ischemia based on non-invasive imaging and clinical history. All aneurysms were in the middle cerebral artery (MCA) territory; seven were in the MCA bifurcation and one in the distal M2. Of the MCA bifurcation aneurysms, three showed complete or near-complete thrombosis at the time of initial diagnosis. One patient harbored multiple intracerebral aneurysms in other locations. Aneurysm diameters ranged from 3 mm to 12 mm (mean diameter: 8, IQR 4.5–10.5 mm). Other potential sources of AIS, including atrial fibrillation and internal carotid artery (ICA) stenosis, were present in five patients.

VWI was performed in six of the eight cases. Of those, four cases showed aneurysm sac enhancement of varying intensities.

### 3.1. Patients with LVO

Of the four patients with LVO, three presented with acute M1 segment occlusion and one with a right terminal ICA occlusion. Only one patient was eligible to receive i.v. thrombolysis, while MT was performed in all four. Successful recanalization was achieved in all patients, and an MCA bifurcation aneurysm was revealed in all cases after reperfusion. Concomitant aneurysm thrombosis, which was not initially identified at the time of stroke, could be retrospectively verified upon the CT, CTA or DSA imaging in three patients. Two patients showed retrograde aneurysm filling on CTA and late-phase CT post-contrast. One patient showed a hyperdensity located outside the expected vessel lumen on non-contrast CT, thus excluding hyperdense artery sign, and aneurysm thrombosis was also seen on DSA. In one patient imaging findings were inconclusive.

Three patients received VWI and of those two showed positive aneurysm wall enhancement. One patient was treated using endovascular coiling a few weeks later and the remaining two were managed conservatively.

### 3.2. Patients Without LVO

Of the four patients without LVO, three of them received VWI and all exhibited positive findings. Two patients, one with a fully and one with a partially thrombosed aneurysm were treated with surgical clipping. No other co-factors for stroke were identified. The other two patients were managed conservatively. One harbored a fully thrombosed aneurysm; the second had no signs of aneurysm thrombosis and was diagnosed with atrial fibrillation and multiple older ischemic lesions in multiple other vascular territories.

**Figure 2:**
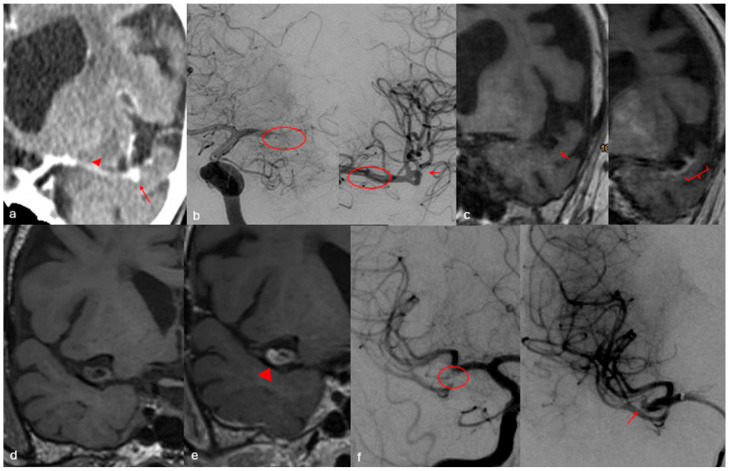
Two cases with parent vessel occlusion. The first case is shown in the upper row. (**a**) Late-phase CT with contrast shows retrograde filling of a left MCA bifurcation aneurysm (red arrow) and a filling defect of the proximal M1 segment (red arrowhead). (**b**) DSA of the left ICA shows an M1 occlusion and complete recanalization after successful mechanical thrombectomy (red circle). The LMCA bifurcation aneurysm is seen after recanalization (red arrow). (**c**) MRI with vessel wall imaging shows no contrast enhancement of the aneurysm wall (red arrow). Cortical enhancement of the left temporal lobe due to subacute ischemic lesions is seen (red bracket). In this case it is improbable that the aneurysmatic lesion was responsible for the stroke. The second case is shown in the lower row where a right MCA bifurcation aneurysm shows intense aneurysm wall enhancement on MRI vessel wall imaging (**d**,**e**) (red arrowhead). T1 sequence without (**d**) and with contrast (**e**) is shown. (**f**) DSA of the right MCA shows occlusion at the origin of the inferior trunk (red circle) with complete recanalization and appearance of the right MCA bifurcation aneurysm (red arrow) after successful aspiration of the clot. In this case the thrombosed aneurysm was regarded as the probable cause of stroke and was coiled at a later date.

**Figure 3:**
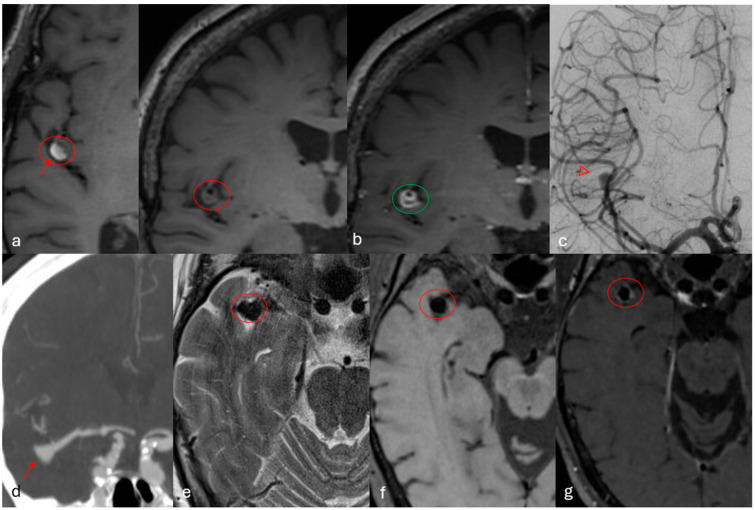
Two cases of patients without large vessel occlusion. (**a**) In the upper row T1 axial and coronal native images show a partially thrombosed aneurysm at the distal M2 segment of the right MCA (red circle). The thrombosed portion with T1 hyperintensity is depicted by the red arrow. (**b**) T1 BB with contrast shows vivid enhancement of the aneurysm wall and clot (green circle). The enhancement is evaluated as vivid because it is intense, well demarcated and close to that of normally enhancing intracranial structures (**c**) Angiographic anteroposterior images show partial contrast filling of the patent part of the sac (open red arrowheads). This case was considered to have a high probability of the aneurysm being responsible for ischemia. The aneurysm was treated surgically with clipping. The second case is depicted in the lower row. (**d**) A CTA shows an inferiorly oriented right MCA bifurcation aneurysm (red arrow). MR imaging with axial (**e**) T2, (**f**) T1 BB and (**g**) T1BB with contrast show a non-thrombosed aneurysm with light contrast enhancement (red circle). The enhancement is evaluated as light because it is subtle, slightly above background parenchyma. The patient was diagnosed with atrial fibrillation, had additional multiple older infarcts in different vascular territories and the cause of the newer ischemic lesions were considered to be unrelated to the aneurysm.

Based on imaging and clinical features, aneurysm-related ischemic events were categorized as: probable when thrombus and enhancement were present with or without stroke risk factors, possible when only thrombus was present with or without stroke risk factors and improbable when stroke risk factors were present with or without enhancement. The two patients with missing VWI, which were currently classified as possible, would have been classified as probable if enhancement was present. Aneurysms classified as a probable cause of stroke were treated either surgically or with an endovascular approach, based on aneurysm morphology, patient age, comorbidities and technical feasibility. In contrast, aneurysms determined to be possible or improbable sources of ischemic events were managed conservatively at the time of diagnosis with more rigorous follow-up imaging to monitor morphological changes or new signs of instability. Imaging findings, treatment, contributing factors, aneurysm characteristics and likelihood are depicted in Table 2. Figure 2 and Figure 3 show illustrative examples of patients with and without LVOs and demonstrate varying degrees of association between aneurysm and ischemic events. There was perfect interobserver agreement between the two independent investigators.

## 4. Discussion

In this observational case series of patients with concomitant cerebral aneurysms in the setting of AIS, we aimed to explore the potential role of aneurysms as a causative or contributing factors to the ischemic event. We analyzed imaging and clinical parameters to identify features suggestive of aneurysm-related stroke. Each case was classified using a three-tiered classification system reflecting diagnostic certainty of probable, possible or improbable. Our evaluation incorporated traditional imaging modalities such as CT, CTA and DSA, along with more advanced MRI techniques, such as VWI sequences. Imaging findings were interpreted in conjunction with clinical history to better characterize the likely etiology of the ischemic events. This study introduces a structured, imaging-based framework which integrates multimodal imaging findings with clinical context to assess the likelihood of a causal relationship between intracranial aneurysm and ischemic stroke.

Amongst the various imaging findings assessed, the presence of complete or partial thrombosis of the aneurysm sac at the time of stroke presentation emerged as one of the stronger predictors. In these cases, thrombosis was considered a plausible source of distal embolization or a potential contributor to local hemodynamic disturbances leading to ischemia. Aneurysm wall enhancement, as shown on VWI was used as an additional predictor of aneurysm instability. Detailed analysis of CT findings, especially in cases with LVOs, enhanced our risk analysis. The anatomical relationship between aneurysm and infarct territory, temporal correlation with symptom onset, and absence of other definitive stroke risk factors also contributed to the likelihood stratification. These observations support a pattern-based approach rather than reliance on isolated imaging findings, allowing for a more detailed assessment of aneurysm-related stroke.

A growing body of literature has documented cases in which unruptured thrombosed or partially thrombosed intracranial aneurysms have been implicated in the development of acute neurologic deficits due to ischemic events. While many of these reports are isolated case studies, they highlight the important mechanisms by which aneurysms may contribute to cerebral ischemia [10,11,12,13]. Several pathophysiological processes have been proposed, including thromboembolic events [10,14,15,16,17], in situ thrombosis with occlusion of perforators [11,18,19], and parent artery occlusion [20,21] being some of the causes. Beyond individual cases reports, larger cohort studies have also identified and included patients where embolic events attributed to the aneurysm were described [1,22,23,24,25,26,27,28].

The prevalence of aneurysms in the general population is estimated to be around 3.2% [29]. In patients with ischemic stroke the prevalence appears to be significantly higher and has been reported to be as high as 9.3% [30]. Furthermore, within the subgroup of patients presenting with LVOs, the prevalence of coexisting aneurysm has been reported to reach up to 5.6% [31]. Although unruptured intracranial aneurysms are not necessarily associated with increased risk of stroke, it is well supported by the literature that aneurysm thrombosis can lead to ischemia [32]. Thrombotic aneurysms at the time of diagnosis should be regarded as hemodynamically and structurally unstable lesions with an inherent propensity to grow, cause thromboembolic events and, in some cases, even rupture [33]. The presence of intraluminal thrombus is often considered a marker of aneurysm instability, because it reflects disturbed aneurysm flow dynamics, altered wall stress and ongoing pathological remodeling within the aneurysm sac [34].

Spontaneous aneurysm thrombosis is a well-documented phenomenon and appears to be more prevalent in larger lesions, but acute parent artery occlusion is extremely rare [35]. However, once it happens, it can lead to malignant cerebral infarction, associated with catastrophic consequences. It often involves larger vascular territories with the potential of rapid clinical deterioration and severe neurologic outcomes. Possible mechanisms of parent artery occlusion include local extension of luminal thrombi from the sac into the parent vessel leading to progressive intraluminal obstruction, embolus resulting in downstream vessel occlusion, direct mass effect on the parent artery due to its size and wall inflammation leading to reduced arterial patency from external compression [33,36]. These mechanisms are not mutually exclusive and may act synergistically, particularly in large or partially thrombosed lesions.

In cases where a partially or fully thrombosed aneurysm is present, identifying the aneurysm as the etiology of stroke with certainty can be problematic. The diagnostic dilemma becomes more pronounced when the thrombosed aneurysm is located within the same vascular territory as the infarct, but other well-recognized causes of stroke are also present. Patients with atherosclerotic lesions or documented atrial fibrillation would typically be categorized under TOAST classification type 1 or type 2 respectively in regards of stroke etiology. If these patients are found to additionally harbor a thrombosed or partially thrombosed aneurysm in the same arterial distribution of ischemia, identification of the cause becomes unclear. To differentiate among possible etiologies, meticulous image analysis becomes essential. Multimodal imaging from invasive and non-invasive imaging can offer critical insights and information collected from imaging findings can help us further elucidate underlying mechanisms.

In cases of parent artery occlusion, non-contrast CT images should include careful inspection for opacifications or hyperdense structures that lie outside the expected vessel lumen, which may indicate presence of thrombosed aneurysmal material. CTA and particularly late-phase contrasted CT images should be scrutinized to identify contrast opacification of aneurysmatic lesions distal to the occlusion site, from retrograde filling or partial flow preservation. In addition, delayed DSA images with retrograde filling of the vasculature could occasionally reveal subtle findings of retrograde aneurysm opacification or absence of such. Integration of these imaging findings with clinical data can enhance diagnostic confidence and contribute to more accurate identification of the underlying cause. Prospective identification of the presence of an aneurysm, its morphologic characteristics, thrombotic status and anatomical relationship to the infarcted territory holds significant clinical value. Not only does it help elucidate the probable cause of stroke but also has practical procedural implications. Awareness of a thrombosed aneurysm in the vascular path of MT will inform the operating physician of potential risks and might influence interventional strategy, device selection and risk mitigation measures, reducing the likelihood of iatrogenic rupture or re-embolization during the endovascular procedure [31,37].

Additionally, in the diagnostic evaluation of AIS patients with coexisting aneurysms, MR–VWI can be a valuable adjunctive imaging tool, particularly in equivocal cases. It has been shown that arterial wall enhancement on MR–VWI is more frequently observed in unstable intracranial aneurysms. It can be used as an indirect marker of vessel wall inflammation, and although not definitive it can serve as a potential marker of aneurysm instability [38]. Aneurysmatic sac contrast enhancement and concomitant thrombus formation or thromboembolism, when seen in conjunction with partial or complete aneurysm thrombosis can significantly alter the likelihood that an aneurysm is the source of distal embolic stroke, increasing the diagnostic certainty from improbable to probable. In cases with positive VWI, no aneurysm thrombosis and established other stroke risk factors, it is rather improbable for an aneurysm to be responsible for downstream ischemia. The wall enhancement could still be a suggestive sign of aneurysm instability, but it does not necessarily indicate that the aneurysm is actively contributing to thromboembolic phenomena at the time the patient presents with a stroke. Such aneurysms may still require follow-up and risk assessment. The cases shown in Figure 2 and Figure 3 highlight how imaging findings can be combined to improve diagnostic accuracy and guide clinical decision making.

It is yet unknown whether the secondary occlusion of a saccular aneurysm by an embolic thrombus originating from the heart or the more proximal arteries [39] will cause aneurysm wall enhancement after revascularization. If this was true, this would potentially lead to false probability classification.

The MCA, and particularly the MCA bifurcation, is recognized as the most common site for the development of aneurysms with partial or complete thrombosis [40,41]. This predilection could be due to the complex hemodynamics stress and vessel geometry of the specific region. This observation was also confirmed in our case series where most of the aneurysms were located at the MCA bifurcation. These aneurysms were identified in the context of the patient’s ischemic event and classified using our three-tiered diagnostic risk profile, as the probable, possible, or improbable cause of stroke. Cases with lower probability were managed conservatively. A risk-adapted management strategy highlights the potential clinical utility of our classification system in guiding treatment decision and individualized patient care. This management approach aligns with the literature, suggesting that in select cases, the risk of recurrence among patients treated conservatively remains low [4] and some patients can be managed conservatively. In such cases, follow-up imaging in relatively short intervals might be needed to further monitor aneurysm stability. In our case series, the imaging characteristics of the aneurysms treated conservatively have remained unaltered over the course of follow-up, further supporting the safety of a conservative image-guided management approach in selecting patients. Although we consider the findings interesting, this study has limitations. It is a retrospective, single-center study with a small number of patients, which makes it difficult to draw definitive conclusions regarding the casual relationship of aneurysm and ischemic stroke. The proposed classification scheme is descriptive and was derived from qualitative evaluation, with no formal statistical analysis. Due to its retrospective nature, the imaging protocols varied and VWI, which we consider an important component of the imaging algorithm, was missing in two patients. The patient population we included in this study was heterogeneous, consisting of patients with and without LVOs. All patients had MCA territory aneurysms only, which introduces a potential selection bias. Additionally, there was no long-term follow-up available. Another limitation of the study is the lack of a control group with patients with stroke and incidental aneurysms in different vascular territories, which would enhance the importance of certain imaging characteristics.

While our findings support the notion that specific imaging characteristics—particularly aneurysm thrombosis and wall enhancement—may be associated with aneurysm-related ischemic events, larger prospective studies are required to validate this proposed framework and further clarify causality.

## 5. Conclusions

In patients presenting with AIS who are also found to have concomitant unruptured intracranial aneurysms, a thorough and integrated imaging approach is essential for evaluating the potential causal relationship between the aneurysm and the ischemic event. Apart from ruling out other causes of stroke, further investigation is needed with detailed observation of traditional imaging along with advanced MRI sequences like VWI. Such an approach can help stratify the probability of an aneurysm being the potential source of the thromboembolic events. This probability-based stratification supported by imaging and clinical characteristics can be highly informative for individualized treatment planning and can facilitate risk-adapted decision making for both acute management and long-term surveillance. The proposed practical framework does not represent a validated diagnostic tool, rather a suggestion of imaging and clinical history interpretation that warrants further investigation.

## Figures and Tables

**Table 1:** Patient demographics and clinical presentation.

Pt	Age	Gender	Clinical Presentation	Stroke Age	NIHSS Admission	NIHSS Discharge
1	83	M	Acute left-sided hemi-syndrome, dysarthria, left-sided neglect in wake-up constellation	Acute	10	0
2	57	F	Dysarthria, word-finding difficulties, anopsia of the lower right quadrant	Subacute—1 month prior TIA with similar presentation	3	0
3	44	F	Slight left-sided weakness, dysarthria in wake-up constellation	Acute	4	0
4	80	F	Right-sided hemisyndrom	Acute—older cerebellar ones	23	2
5	71	F	Moderate weakness in left leg	Subacute	2	1
6	64	F	Mouth droop, arm weakness (left), dysarthria	Acute—old from previous M2 occlusion	3	1
7	74	F	Left-sided hemiparesis, dysarthria, hypoasthesia	Acute—multiple older ones in other territories	7	6
8	80	F	Left-sided hemiparesis, dysarthria, anisocoria	Acute	12	1

**Table 2:** Aneurysm characteristics, imaging findings, related ischemic event likelihood and treatment.

Pt	Location	Thrombus	Size	LVO	VWI	Treatment	Co-Factors	Classification Parameters		Aneurysm-Related Ischemic Event
1	RMCA bifurcation	Near-complete	7 mm	Yes M1	+	Coiling	RICA stenosis	Thrombus Enhancement Stroke risk factor	+ + +	Probable
2	LMCA bifurcation	Near-complete	11 mm	no	+	Clipping	No	Thrombus Enhancement Stroke risk factor	+ + −	Probable
3 *	RMCA bifurcation	Unknown—imaging inconclusive	3 mm	Yes M1	N/A	Conservative	No	Thrombus Enhancement Stroke risk factor	+/− N/A −	Possible
4	LMCA bifurcation	No thrombus—CT shows retrograde aneurysm filling	4.5 mm	Yes M1	−	Conservative	Atrial fibrillation/older cerebellar infarcts on the left	Thrombus Enhancement Stroke risk factor	− − +	Improbable
5	RMCA distal M2	Partial	10 mm	no	+	Clipping	No	Thrombus Enhancement Stroke risk factor	+ + -	Probable
6 *	RMCA bifurcation	Complete	12 mm	no	N/A	Conservative	Cavernous ICA stenosis/older M2 occlusion	Thrombus Enhancement Stroke risk factor	+ N/A +	Possible
7	RMCA bifurcation	No	7 mm	no	+	Conservative	Atrial fibrillation/multiple older ischemic lesions in other vascular territories	Thrombus Enhancement Stroke risk factor	− + +	Improbable
8	RMCA bifurcation	No	4.5 mm	Yes ICA	+	Conservative	Atrial fibrillation	Thrombus Enhancement Stroke risk factor	− + +	Improbable

* Cases with asterisk are considered lower certainty classifications due to absence of VWI. + means positive/present and − means negative/absent.

## Data Availability

Data are available upon request from Z.K.
